# Physical function and activity, pain, and health status in adults with myelomeningocele after orthotic management from childhood: a descriptive study

**DOI:** 10.1186/s12891-023-06673-7

**Published:** 2023-07-03

**Authors:** Åsa Bartonek, Marie Eriksson

**Affiliations:** Division of Paediatric Neurology, Department of Women’s and Children’s Health, Karolinska Institutet, Karolinska University Hospital, Motoriklab QA: 27, Karolinska vägen 37 A, S-17176 Stockholm, Sweden

**Keywords:** Ambulation, Functional capacity, MMC, Orthoses, Sit-to-stand, Spina bifida

## Abstract

**Background:**

Individuals with myelomeningocele (MMC) exhibit neurological deficits below the lesion level involving both motor and sensory functions. Ambulation and functional outcomes in patients offered orthotic management since childhood were investigated.

**Methods:**

Physical function, physical activity, pain, and health status were assessed in a descriptive study.

**Results:**

Of 59 adults with MMC, aged 18–33 years, 12 were in the community ambulation (Ca), 19 in the household ambulation (Ha), six in the non-functional (N-f), and 22 in the non-ambulation (N-a) groups. Orthoses were used by 78% (*n* = 46), i.e., by 10/12 in the Ca, 17/19 in the Ha, 6/6 in the N-f, and 13/22 in the N-a groups. In the ten-metre walking test, the non-orthosis group (NO) walked faster than those wearing ankle-foot orthoses (AFOs) or free-articulated knee-ankle-foot orthoses (KAFO-Fs), the Ca group faster than the Ha and N-f groups, and the Ha group faster than the N-f group. In the six-minute walking test, the Ca group walked farther than the Ha group. In the five times sit-to-stand test, the AFO and KAFO-F groups required longer than the NO group, and the KAFO-F group longer than the foot orthosis (FO) group. Lower extremity function with orthoses was higher in the FO than the AFO and KAFO-F groups, higher in the KAFO-F than the AFO group, and higher in the AFO group than in those using trunk-hip-knee-ankle-foot orthoses. Functional independence increased with ambulatory function. Time spent in physical recreation was higher in the Ha than the Ca and N-a groups. There were no differences between the ambulation groups in rated pain or reported health status.

**Conclusion:**

The physical function results in persons with MMC improve our understanding of this population’s heterogeneity and shed light on the importance of individualized orthotic management. The similarities between the various ambulatory levels in physical activity, pain, and health status may mirror opportunities to achieve equal results regardless of disability level. A clinical implication of the study is that orthotic management is likely to be beneficial for the patient with MMC of which the majority used their orthoses for most time of the day.

## Background

The most clinically significant subtype of spina bifida (SB) is myelomeningocele (MMC), a condition characterized by failure of the spinal neural tube to close during embryonic development, resulting in neurological deficits that vary with the lesion level. Individuals with MMC typically exhibit neurological deficits below the lesion level, involving both motor and sensory functions [1]. In persons with MMC, cooperation between many specialists is crucial, with lifelong follow-up services and social support required due to the complex set of physical, cognitive, and social needs [2, 3]. To compensate for muscle pareses, orthoses are used, provided by the certified prosthetist/orthotist (CPO), often in collaboration with orthopaedists and physiotherapists. Since the 1990s, active rehabilitation of children with MMC in Sweden has started from early childhood, with orthotic management intended to support each child’s potential motor function [4, 5]. The patients in this study grew up with this orthotic concept and were offered management by the same orthotic provider from childhood to adulthood. Some patients used an energy-storing carbon fibre orthosis since 2000, which has been shown to improve gait kinetics and spatiotemporal aspects of gait in some patients [6].

Based on the manual muscle testing of quadriceps [7], grades 4 and 5 (against resistance to normal) have been defined as low-level neurological lesion and grade 3 or below (against gravity or less) as high-level neurological lesion [8, 9]. For a patient to walk brace free or with an ankle-foot orthosis, the quadriceps muscle must be at least Grade 4 or 5 in strength [10]. Additionally, the same authors stated that patients should be classified according to whether their limbs are spastic or flaccid in addition to grouping them according to the patterns of muscle weakness and paralysis. This was strengthened by findings that children with MMC with similar muscle paresis exhibited different ambulatory functions, with approximately half achieving the expected ambulation considered possible according to their motor paresis [11].

Change in transfer ability is reportedly directly related to a corresponding change in motor level, possibly explained by changes in the muscle strength of the iliopsoas and quadriceps [12]. Recent findings confirm that effects on the ambulation status of commonly accepted clinical factors in persons with MMC, such as motor level, vary with time and that account should be taken of the time-varying nature of clinical reality, for example, during voluntarily transitioning to wheelchair use with increasing demands for physical mobility in teenage years [13]. From a large study of individuals with MMC or meningocele aged 16–80 years (mean 34 years), a history of shunting for hydrocephalus, higher motor level, and a history of surgical release of hip or knee contractures were found to be associated with ambulatory status [14]. In a cohort of SB cases followed over 50 years, mobility declined with age, possibly because of increasing obesity and deteriorating health [15]. In persons with SB older than 50 years, obesity, hypertension, pain, and deterioration in ambulatory function were found even in well-functioning SB cases [16], and SB severity and Body mass index (BMI) were found to be the most influential factors in those with early-onset deterioration in gait function [17]. Alriksson-Schmidt et al. [18] reported on general health perceived today in adults with MMC using the EQ-5D-5 L visual analogue scale (VAS) [19], considering that individuals with MMC are at high risk of poor health and emphasizing the importance of both subjective and objective measures of health. Moreover, pain was frequently found in the same study, with women being more likely to report that pain interfered with work than were men [18].

Bloemen et al. [20] reported that older age and inability to walk negatively influenced physical activity in children 5–19 years old diagnosed with SB and who were wheelchair users. Adolescents and young adults with MMC are reportedly physically inactive, mainly related to aerobic fitness in non-ambulatory persons [21], with gender and ambulatory status found to be important determinants of oxygen uptake, which was somewhat related to muscle strength [22], and with sport participation being associated with high self-reported physical activity [23]. Additionally, cardiovascular disease risk factors were identified in a large proportion of adolescents and young adults with MMC [24].

In adult cohorts with MMC, 75% wore orthoses of some kind [2] and 14% used orthoses for walking [3]. In the study of Dicianno et al. [14], 33% of participants used orthoses, although this was not related to the level of paresis or walking function. The aim of the present study was to explore outcomes with respect to ambulation in physical function and physical activity in adult persons with MMC who used orthoses and had received orthotic management from early childhood. Further questions concerned the participants’ pain perception and health status. Ethical approval for the study was obtained from the Regional Ethical Review Board in Stockhom, Sweden (Dnr 2017/910 − 31/4), and written informed consent was obtained from all participants.

## Methods

Of 91 adult persons with MMC who were contacted, 59 agreed to participate in the study. Nine persons were available through telephone contact. Inclusion criteria were age of 18 years, having been born in or after 1985, and having used orthoses since childhood as prescribed by the same local orthotic provider. Twenty-five (42%) of the participants were females and the age was mean (standard deviation [SD]), 25.8 (4) years. The sociodemographic variables household status, main occupation, and wheelchair use were documented. Based on a muscle strength examination [7], the neurological levels were designated in terms of muscle function classes (MFC) I–V [11]. Spasticity in the lower limb muscles and presence of pressure sores were documented. Information on shunted hydrocephalus was collected from medical records. BMI was calculated based on body weight and height. Extensive information about this study population has been published elsewhere (manuscript under review).

Types of orthoses were grouped in accordance with the international classification (ISO 2007) [25] as foot orthosis (FO), ankle-foot orthosis (AFO), knee-ankle-foot orthosis (KAFO), and hip-knee-ankle-foot orthosis (HKAFO). KAFOs were specified as KAFO-F (free-knee articulating) [26] and KAFO-L (locked knee joint). Trunk-hip-knee-ankle-foot orthoses (THKAFOs) [27] including a trunk segment were added. Participants not using orthoses were designated the non-orthosis group (NO).

Participants able to walk outdoors were assigned to the community ambulation group (Ca), those walking indoors to the household ambulation group (Ha), those walking only a limited time in therapy session to the non-functional ambulation group (N-f), and those using a wheelchair for all transfers to the non-ambulation (N-a) group [28]. Use of walking aids was registered.

### Physical function assessment

The ten-metre-walk test (10MWT) was used to measure walking speed in metres per second over a short distance; with test repetition, it is a reliable measure of gait velocity in ambulatory adult patients at both fast and self-selected speeds [29]. The participants were instructed to walk at their fastest pace twice.

The five times sit-to-stand (5STS) protocol was used to evaluate the time taken to stand from a seated position [30]. The participants were instructed to rise from a bench without armrests five times as fast as possible with their arms folded across their chests. A frame was located approximately half a metre in front of the participants for security. All participants used the same bench with a seat height of 44.5 cm. The test was performed twice and timed to a hundredth of a second.

The submaximal level of functional capacity was assessed with the self-paced six-minute walk test (6MWT), measuring the distance in metres walked in six minutes [31] on an oval track 22 m long with or without a walking aid. The participants were allowed to take a short break by standing still if required. Participants who could not perform the 6MWT performed the six-minute push test (6MPT) with a manual wheelchair [32]. Before and immediately after completion of the 6MWT and the 6MPT, the participants were requested to estimate physical exertion on a Borg scale [33].

Functional status with orthoses was assessed with the Swedish version of the Orthotics and Prosthetics Users’ Survey – Lower Extremity Functional Status (OPUS-LEFS) [34, 35]. The participants answered 24 questions regarding ease or difficulty of managing everyday functions based on five alternatives ranging from “very easy” to “cannot do this activity”. They were also asked whether they typically wore an orthotic device to perform the activity.

The participants’ functional independence was assessed with the Functional Independence Measure (FIM) [36] using the motor subscale covering eating, grooming, bathing, dressing upper body, dressing lower body, toileting, bladder management, bowel management, transfers (i.e., bed/chair/wheelchair, toilet, and tub/shower), and locomotion (i.e., walk/wheelchair and stairs). Each item is scored on a seven-point ordinal scale ranging from 1 to 7, with higher scores indicating more independence.

### Physical activity

Current physical activity (PA) status was assessed using a questionnaire [37] covering “recreation” activities, such as staying outdoors or taking a wheelchair trip, “exercise”, i.e., planned activities to maintain or improve physical fitness, and “sport”, involving physical exertion often undertaken competitively.

### Pain

Perceived pain on the examination day and during the preceding four weeks was assessed using a vertical visual analogue scale (VAS) [38]. If pain was present, the participants were asked to mention the location verbally. They were also asked to illustrate the location by drawing on a pre-printed figure on a paper [39].

### Health status

Health status was documented using the EQ-5D-5 L, which is a generic instrument measuring health status and consisting of two parts. The first assesses health in five dimensions (i.e., mobility, self-care, usual activities, pain/discomfort, and anxiety/depression), each of which has five response levels (i.e., no problems, slight problems, moderate problems, severe problems, and extreme problems/unable to). The participant was requested to indicate his/her health state by checking the box next to the most appropriate response level for each of the five dimensions. In the second part, the participant rated his/her perceived health using the EQ-5D-5 L VAS, ranging from 0 (“worst imaginable health”) to 100 (“best imaginable health”) [19].

### Data and statistical analysis

Descriptive data are presented as mean and SD, median, and minimum/maximum (min/max) values. The Kruskal–Wallis test with the post hoc Mann-Whitney test was used to analyse use of walking aids, 10MWT, 5STS, 6MWT, 6MPT, physical exertion according to Borg score, FIM motor score, pain, and VAS Health status score with respect to ambulation groups. One-way analysis of variance (ANOVA) with Bonferroni post hoc adjustment was used to analyse differences in OPUS-LEFS between ambulation groups. Spearman rank correlation coefficients were used to explore associations between various factors. Correlations were considered significant when *p* < 0.05. Correlations were interpreted as follows: correlation coefficient 0.00–0.20 as slight, 0.21–0.40 as fair, 0.41–0.60 as moderate, 0.61–0.80 as substantial, and 0.81–1.00 as almost perfect correlation [40]. Statistical analyses were carried out using SPSS version 28.0. The significance level was set at *p* < 0.05.

## Results

**Fig. 1:**
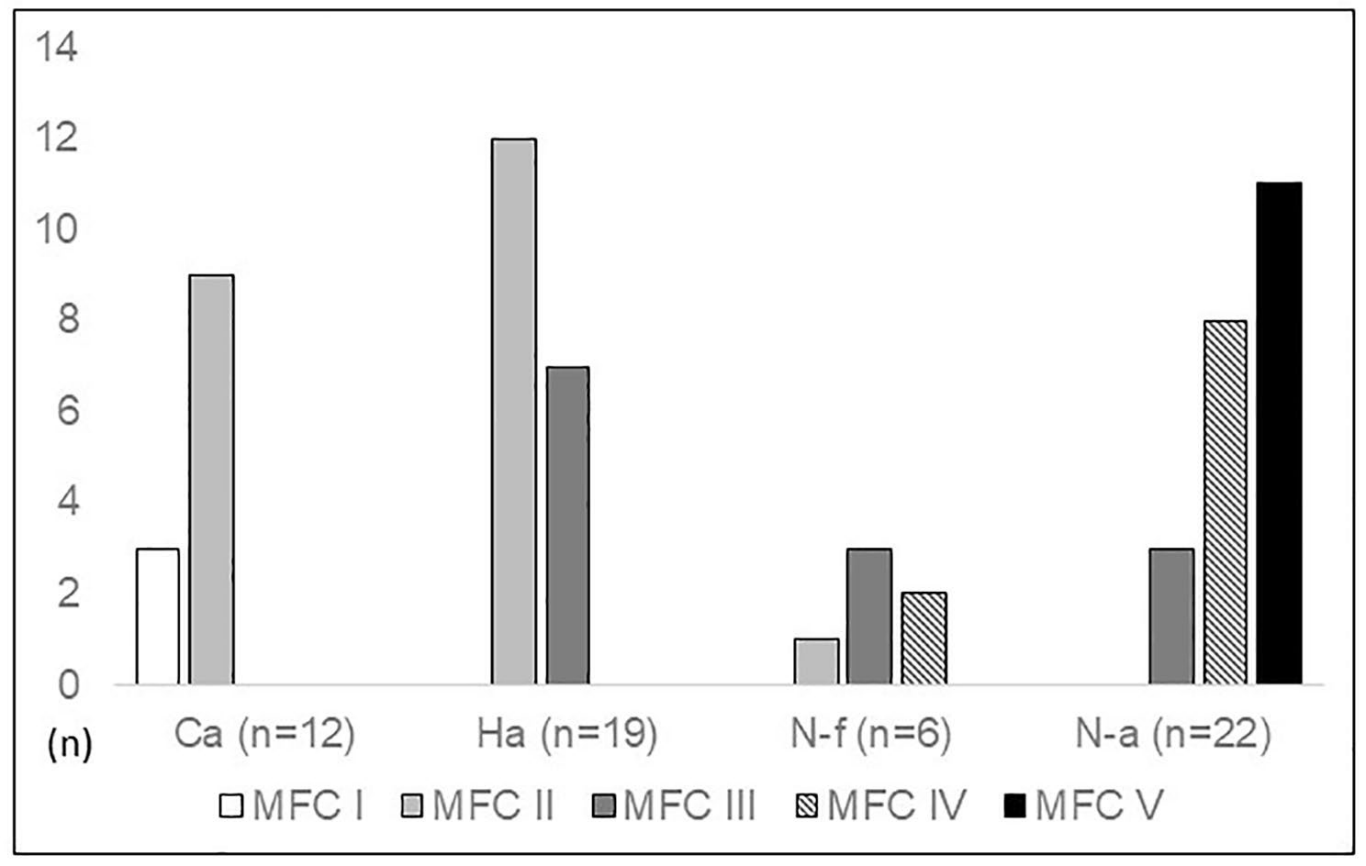
The neurological levels expressed as muscle function classes (MFCs) with respect to ambulation groups (Ca: community ambulation; Ha: household ambulation; N-f: non-functional ambulation; N-a: non-ambulation)

Ambulatory function was present as Ca in 12 participants, as Ha in 19, as N-f in six, and as Na in 22 participants. Of the entire study group, 26 (44%) participants lived with parents, three (5%) with a partner, 26 (44%) in a single-person household, and four (7%) in sheltered living. The main occupation was schooling/education in 16 participants (27%), full-time work in 12 (20%), part-time work in 11 (19%), unemployment in four (7%), protected work in five (8%), and pensioned in three (5%) participants. Eighteen (31%) participants had a driving licence. Fifty-three (90%) had shunted hydrocephalus, 34 (58%) had spasticity in the lower limbs, and three (5%) had a pressure sore. Weight and height were significantly lower in the N-a than the Ca group (*p* = 0.017 and < 0.001, respectively) and height was lower in the N-f than the Ca group (*p* = 0.050). Table 1 shows the age, gender, weight, height, BMI, household status, main occupation, driving licence status, presence of shunted hydrocephalus, spasticity, and pressure sore status with respect to ambulation groups. In those nine participants from whom data were collected via telephone interview, the neurological lesions and presence of spasticity were known by the examiner. The neurological levels in terms of MFC with respect to ambulation group are illustrated in Fig. 1.

**Table 1:** Age, gender, weight, height, body mass index (BMI), household status, main occupation, driving licence, presence of shunted hydrocephalus, spasticity, and pressure sore with respect to ambulation groups

	Ca (*n* = 12)	Ha (*n* = 19)	N-f (*n* = 6)	N-a (*n* = 22)	*p*
Age (years) Mean (SD) (min, max)	26.2 (4.2) (19.8, 32.5)	25.6 (3.7) (18.1, 32.7)	29,0 (0.2) (25.6, 32.6)	24.9 (4.4) (18.2, 33.2)	0.171
Gender (female, male)	f: 7, m: 5	f: 5, m: 14	f: 3, m: 3	f: 10, m: 12	0.323
Weight (kg)^a^ Mean (SD) (min, max)	73.9 (16.4) (55.0, 103.0)	69.0 (14.2) (53.4, 107.3)	69.0 (14,0) (46, 82.6)	57.0 (12.4) (35.0, 85.0)	**0.034**
Height (cm)^a^ Mean (SD) (min, max)	166 (9) (157, 184)	160 (8) (140, 178)	155 (7) (147, 167)	146 (11) (125, 171)	**< 0.001**
BMI^a^ Mean (SD) (min, max)	26.8 (6.5) (20.3, 39.7)	26.8 (5.4) (20.1, 41.4)	28.9 (8) (16.5, 34.8)	26.9 (6.3) (18.1, 42.2)	0.815
Household status^b^					0.443
With parents	5	8	0	13	
With partner	1	1	1	0	
Single household	5	9	4	8	
Sheltered living	1	1	1	1	
Main occupation^b^					0.458
School/education	4	5	3	4	
Work full time	4	6	0	2	
Work part time	2	3	0	6	
Unemployed	1	3	3	6	
Protected work	1	1	0	2	
Pensioner	0	1	0	2	
Driving licence^b^	6	6	0	6	0.179
Shunted hydrocephalus ^b^	9	16	6	22	0.086
Spasticity in lower limbs^b^	4	11	4	15	0.250
Pressure sore^b^	0	2	0	1	0.423

Ca: community ambulation, Ha: household ambulation, N-f: non-functional ambulation, N-a: non-ambulation

^a^ Data lacking for those participating in telephone interview

^b^ Number of participants

Bolded text indicates statistical significance

**Fig. 2:**
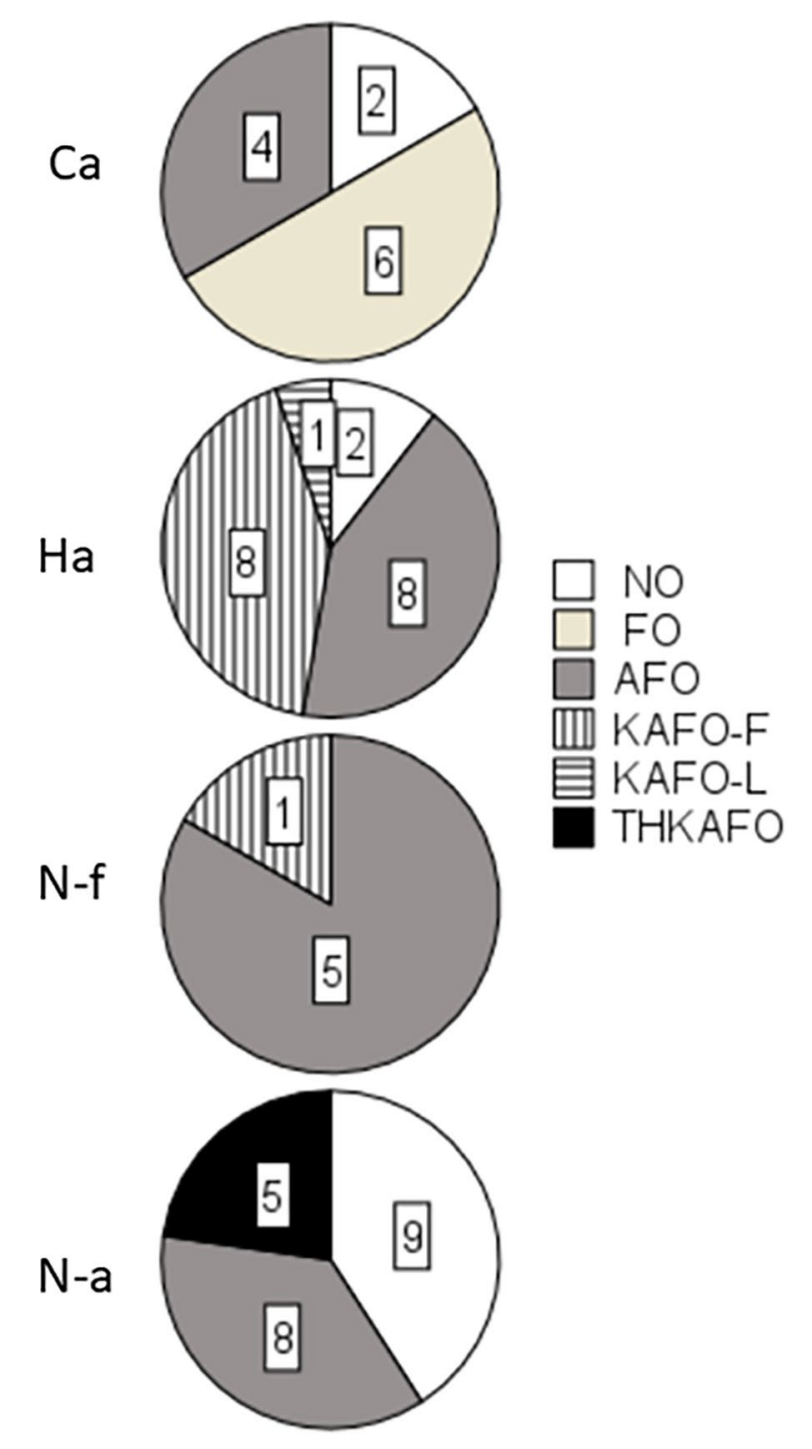
Orthosis use in the groups not wearing orthoses (NO), use of foot orthoses (FO), knee-ankle-foot orthoses (AFO), knee-ankle-foot orthoses with free knee joint (KAFO-F), knee-ankle-foot orthoses with locked knee joint (KAFO-L), and trunk-hip-knee-ankle-foot orthoses (THKAFO with respect to ambulation groups (Ca: community ambulation; Ha: household ambulation; N-f: non-functional ambulation; N-a: non-ambulation)

Orthoses were used by 46 (78%) (Fig. 2). Forty-one/46 (89%) participants used their orthoses 10 or more hours per day, of whom 10 (100%) participants were in the Ca, 16 (94%) in the Ha, six (100%) in the N-f, and nine (69%) in the N-a groups. Energy-storing carbon-fibre spring orthoses [6] were used by two participants in the Ca and nine in the Ha groups.

### Physical function

Table 2 shows the results for use of walking aids and wheelchairs, 10MWT, 5STS, 6MWT, 6MPT, physical exertion change, functional status with orthoses (OPUS-LEFS), FIM, PA, and pain with respect to ambulation groups.

**Table 2:** Use of walking aid and wheelchair, ten-metre walking test (10MWT), five times sit-to-stand (5STS), six-minute walking test (6MWT), six-minute push test (6MPT), physical exertion change, functional status with orthoses (OPUS-LEFS), functional independence (FIM), functional independence score of 78 (FIM 78), physical activity (PA), pain location, and EQ-5D-5 L VAS score with respect to ambulation groups

	Ca (*n* = 12)		Ha (*n* = 19)		N-f (*n* = 6)		Na (*n* = 22)		*p*
	*n* ^a^		*n* ^a^		*n* ^a^		*n* ^a^		
Walking aid^b^	12	0	19	2	6	6	0	-	**< 0.001**
Wheelchair, manual^b^	12	0	19	17	6	6		22	**< 0.001**
Powered self-transportation^b^	12	0	19	10	6	2		16	**< 0.001**
10MWT (m/sec) median (min, max)	9	1.67 (1.21, 2.58)	17	2.22 (1.36, 6.61)	3	3.89 (2.93, 4.08)	0	-	**0.005**
5STS (sec) median (min, max)	8	10.2 (6.9, 14.8)	16	14.4 (7.6, 32.2)	4	15.1 (9.3, 20.2)	0	-	0.081
6MWT (m) median (min, max)	9	445 (316, 504)	9	333 (209, 421)	1	154	0	-	**0.015**
6MWT Physical exertion change median (min, max)	9	5 (0,10)	9	8 (4,11)	1	5	0	-	0.090
6MPT (m) median (min, max)	0	-	6	467 (264, 564)	5	418 (396, 560)	15	462 (267, 616)	0.984
6MPT Physical exertion change median (min, max)	0	-	6	5.5 (1, 8)	5	5 (1, 10)	15	7 (2, 13)	0.476
OPUS-LEFS Mean (SD) (min, max)	10	66.9 (12.9) (45.7, 91.4)	17	46.7 (6,5) (34.1, 54.5)	6	35.4 (7.2) (24.8, 43.2)	13	22.8 (4.3) (17.1, 30.8)	**< 0.001**
FIM motor score median (min, max)	12	87 (83, 89)	19	81.0 (41, 89)	6	81.0 (53, 87)	22	60.0 (27, 81)	**< 0.001**
FIM 78^b^	12	12	19	14	6	4	22	4	**< 0.001**
PA^c^ Recreation^a^	12	6 (50%)	19	16 (84%)	6	5 (83%)		13 (60%)	0.144
PA^c^ Recreation (min/week) median (min, max)	12	22.5 (0, 140)	19	100 (0, 420)	6	80 (0, 210)		30 (0, 300)	**0.012**
PA^c^ Exercise^b^	12	6 (50%)	19	14 (74%)	6	3 (50%)	22	17 (77%)	0.289
PA^c^ Exercise (min/week) median (min, max)	12	10 (0, 180)	19	120 (0, 300)	6	75 (0, 270)	22	110 (0, 570)	0.079
Sport^b^	12	0	19	3 (16%)	6	1 (17%)	22	3 (14%)	0.560
Pain^b^	12	6	19	12	6	5	22	12	0.541
Back		2		8		3		6	
Neck/head		1		2		0		2	
Hips		2		3		0		4	
Knees		1		2		0		0	
Feet		0		1		0		0	
Shoulder		2		2		3		6	
Arms		0		0		0		1	
Hands		0		0		0		2	
Other: Headache				1					
Other: Nerve pain				2					
Other: Pelvis						1			
Pain EQ-5D-5 L VAS score	6	60 (7, 85)	12	50 (16, 95)	5	70 (10, 85)	12	55 (9, 98)	0.972

Ca: community ambulation, Ha: household ambulation, N-f: non-functional ambulation, N-a: non-ambulation

^a^ Number of participants involved in each method

^b^ Number of participants

^c^ PA: physical activity

Bolded text indicates statistical significance

Walking aids were used by eight (14%) participants. Forty-five (76%) participants used a manual wheelchair and 28 (47%) powered transportation. Manual wheelchairs were more frequently used in the Ha, N-f, and N-a groups than in the Ca group (all *p* < 0.001) and powered self-transportation was more frequently used in the Ha and N-a groups than in the Ca group (*p* = 0.014 and *p* < 0.001, respectively).

The 10MWT was performed by 29 participants, of whom seven used walking aids. Time to perform 10MWT differed significantly between the ambulation groups (Table 2). The Ca group walked faster than did the Ha group (*p* = 0.016) or the N-f group (0.009), and the Ha group walked faster than did the N-f group (*p* = 0.040). The NO group (n = 3) walked faster than did the AFO (n = 11) and KAFO-F (n = 9) groups, i.e., median (min, max) metres/second 2.04 (1.72, 2.29) versus 1.38 (0.71, 1.76) (*p* = 0.011) and 1.10 (0.42, 1.69) (*p* = 0.009), respectively.

**Fig. 3:**
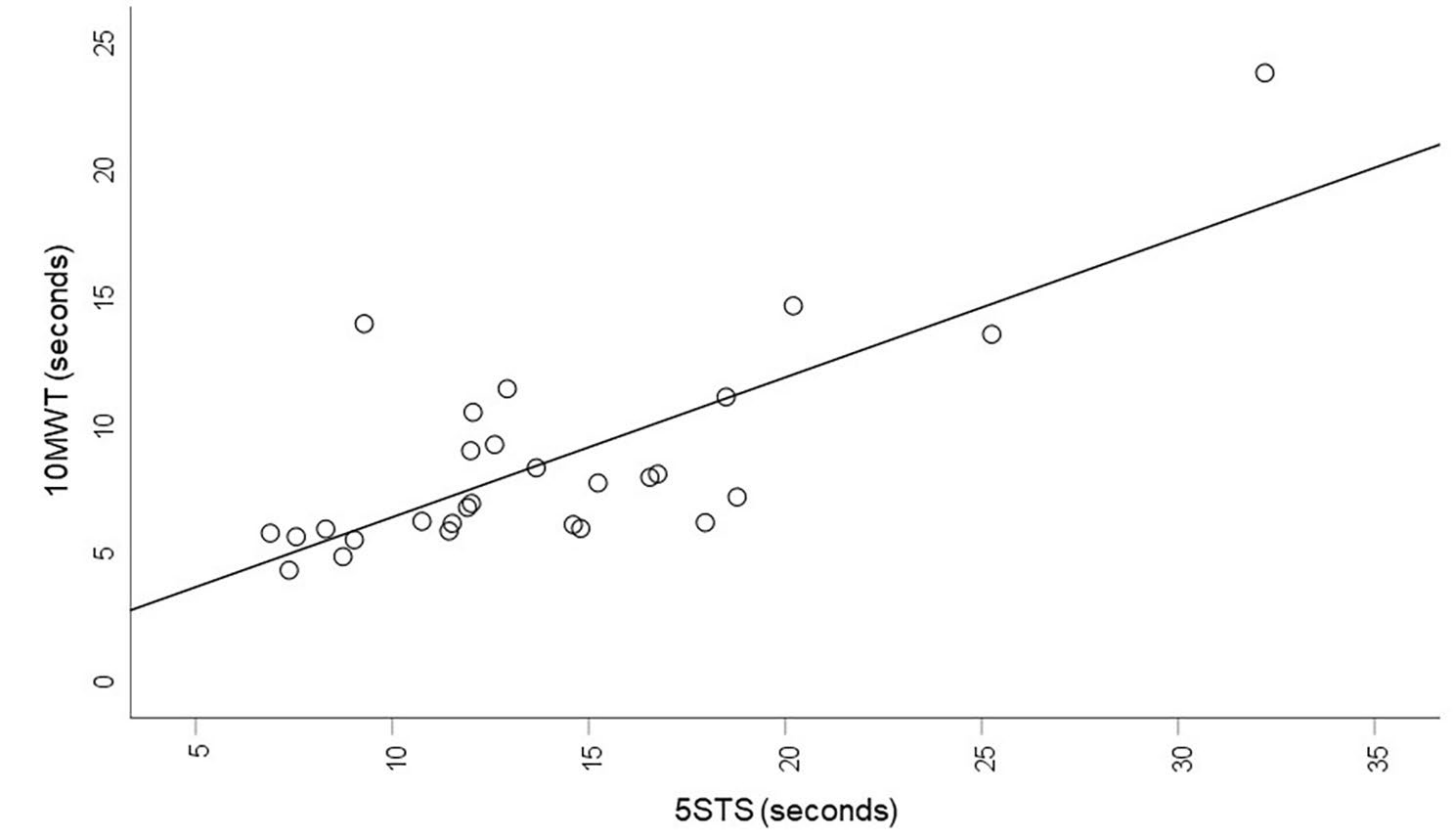
Correlation between ten-metre walking test (10MWT) and five times sit-to-stand test (5STS) presented in seconds for the ambulation groups Ca (community ambulation), Ha (household ambulation), and N-f (non-functional) together

The 5STS test was performed by 29 participants. Time to perform 5STS increased from the Ca to N-f groups, but not significantly (Table 2). The AFO (n = 12) and KAFO-F (n = 9) groups required longer to perform the 5STS test than did the NO group (n = 3), i.e., median (min, max) seconds 12.86 (7.56, 18.78) and 16.76 (11.45, 32.21) versus 7.38 (6.90, 8.75) (*p* = 0.009 and 0.009, respectively) and the KAFO-F group required longer than the FO group (n = 5), i.e., 11.9 (8.31, 14.61) (*p* = 0.029). There was a substantial positive correlation between 10MWT and 5STS presented in seconds for the Ca, Ha, and N-f groups taken together, i.e., median 7 s (min/max 4/24) versus 13 s (min/max 7/32) (*p* < 0.001, Spearman rank correlation coefficient 0.675 s; Fig. 3).

Nineteen participants completed the 6MWT (Table 2). The Ca group walked farther than did the Ha group (*p* = 0.011). Twenty-six participants performed the 6MPT (Table 2). There was no difference in the increase in scored physical exertion between those performing 6MWT and 6MPT, i.e., median (min, max) points 6 (0, 11) versus 6 (1, 13) (*p* = 0.451).

**Fig. 4:**
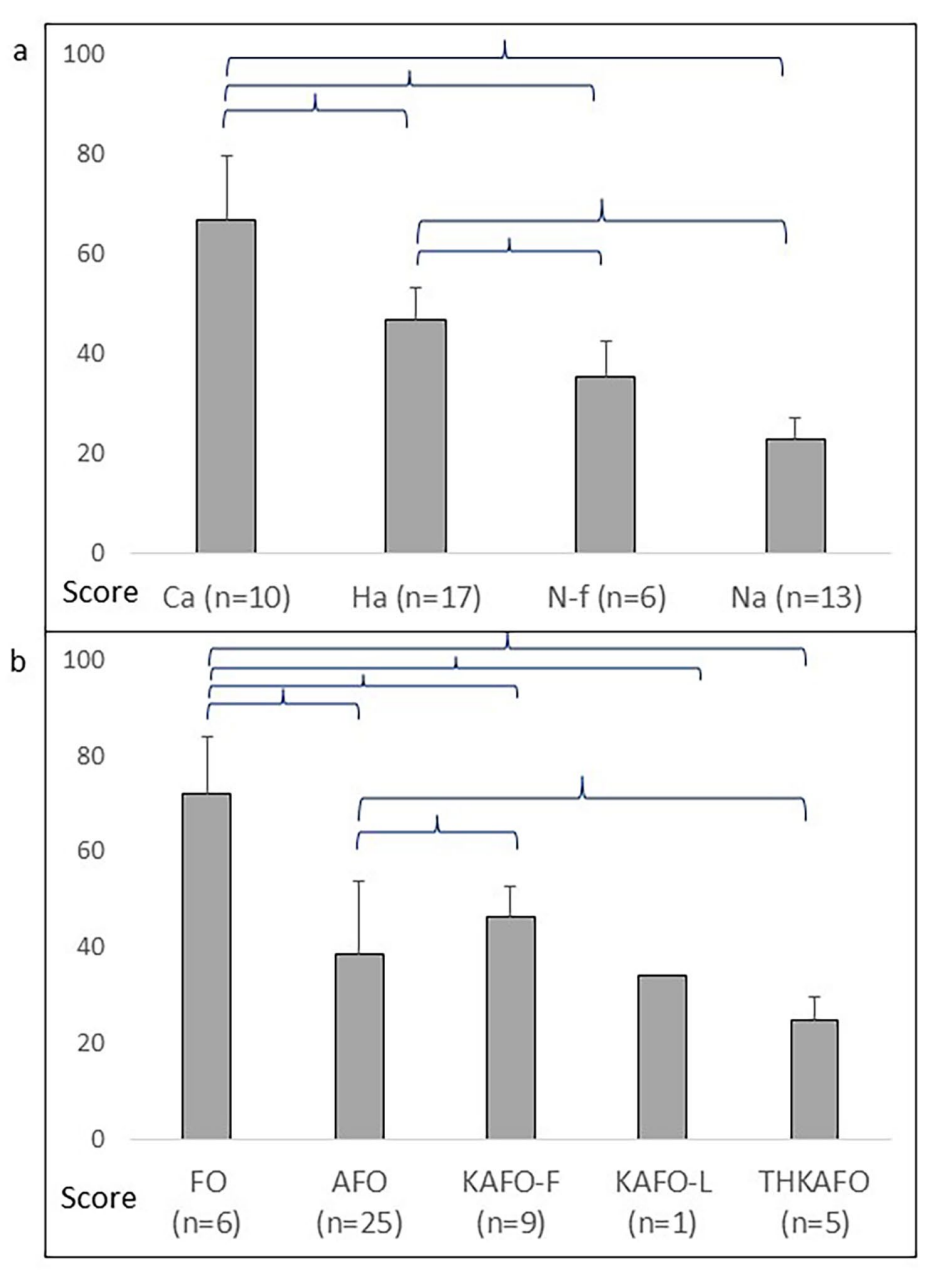
Function with orthoses assessed with OPUS-LEFS with respect to: **(a)** the ambulation groups Ca (community ambulation), Ha (household ambulation), N-f (non-functional ambulation), and N-a (non-ambulation); **(b)** orthosis use in the groups of foot orthoses (FO), knee-ankle-foot orthoses (AFO), knee-ankle-foot orthoses with free knee joint (KAFO-F), knee-ankle-foot orthoses with locked knee joint (KAFO-L), and trunk-hip-knee-ankle-foot orthoses (THKAFO).

Functional status in terms of OPUS-LEFS was assessed for those 46 participants who used orthotic devices (Table 2). The Ca group (n = 10) scored higher than did the Ha (n = 17), N-f (n = 6), and N-a (n = 13) groups, i.e., mean (SD), 66.8 (12.9) versus 46.7 (6.4) (*p* < 0.001), 35.3 (7.1) (*p* < 0.001), and 22.8 (4.3) (*p* < 0.001), respectively. The Ha group scored higher than did the N-f (*p* = 0.026) and N-a (*p* < 0.001) groups, and the N-f group scored higher than did the N-a group (*p* = 0.015) (Fig. 4a). The OPUS-LEFS score was: higher in the group wearing FOs (n = 6) than in the groups wearing AFOs (n = 25), KAFO-Fs (n = 9), and THKAFOs (n = 5), i.e., mean (SD), 71.9 (12.2) versus 38.4 (15.4) (*p* < 0.001), 46.6 (6.1) (*p* = 0.001), and 24.9 (4.8) (*p* < 0.001), respectively; higher in the group wearing KAFO-Fs than in the group wearing AFOs (*p* = 0.035); and higher in the group wearing AFOs than in the group wearing THKAFOs (*p* = 0.002) (Fig. 4b). Table 3 shows orthosis use in 46 participants in activities according to OPUS-LEFS. In item 20, the question about whether or not the orthosis was used was considered inapplicable.

**Table 3:** Use of orthoses in 46 participants with myelomeningocele in activities according to Lower Extremity Functional Status (LEFS) scale of OPUS.

		Ca (*n* = 10)		Ha (*n* = 17)		N-f (*n* = 6)		N-a (*n* = 13)	
	Orthosis type	FO: 6 AFO:4		AFO: 8 KAFO-F: 8 KAFO-L: 1		AFO: 5 KAFO-F: 1		AFO: 8 THKAFO: 5	
Item	Activity	*n*	%	*n*	%	*n*	%	*n*	%
1	Get into/out of tub or shower	0	0	3	17.6	0	0	0	0
2	Dress lower body	0	0	10	58.8	2	33.3	0	0
3	Get on and off toilet	7	70	17	100	6	100	3	23.1
4	Get up from floor	8	80	16	94.1	6	100	3	23.1
5	Balance while standing (with support)	10	100	17	100	6	100	5	38.5
6	Stand for one-half hour (with support)	9	90	16	94.1	3	50	6	46.2
7	Pick up an object from floor while standing	9	90	16	94.1	2	33.3	0	0
8	Get up from a chair	10	100	17	100	6	100	2	15.4
9	Get into and out of a car	10	100	17	100	5	83.3	4	30.8
10	Walk indoors	10	100	17	100	5	83.3	0	0
11	Walk outdoors on uneven ground	10	100	14	82.4	2	33.3	0	0
12	Walk outdoors in bad weather	10	100	10	58.8	1	16.7	0	0
13	Walk up to two hours	7	70	3	17.6	0	0	0	0
14	Walk up steep ramp	10	100	16	94.1	1	16.7	0	0
15	Get on and off escalator	10	100	14	82.4	2	33.3	0	0
16	Climb one flight of stairs with rail	10	100	16	94.1	4	66.7	0	0
17	Climb one flight of stairs without rail	10	100	10	58.8	0	0	0	0
18	Run one block	9	90	4	23.5	0	0	0	0
19	Carry a plate of food while walking	10	100	13	76.5	1	16.7	0	0
20	Put on and take off orthopaedic assistive device	NA							
21	Stand up for one hour	10	100	7	41.2	1	16.7	2	15.4
22	Go shopping for half a day	8	80	3	17.6	0	0	0	0
23	Stand and walk for half a workday (4–5 h)	8	80	3	17.6	0	0	0	0
24	Stand and walk for a workday (8–9 h)	7	70	2	11.8	0	0	0	0

Ca: community ambulation, Ha: household ambulation, N-f: non-functional ambulation, N-a: non ambulation. FO: foot orthoses. AFO: ankle-foot orthoses, KAFO-F: knee-ankle-foot orthoses, free-articulated, KAFO-L: knee-ankle-foot orthosis, locked, THKAFO: trunk-hip-knee-ankle-foot orthosis

NA: Not applicable

Functional independence was assessed with the motor subscale of FIM in 59 participants (Table 2), of whom the Ca group had a higher FIM score than did the Ha (*p* < 0.001), N-f (*p* = 0.002), and N-a (*p* < 0.001) groups, and the N-f group a higher score than did the N-a group (*p* = 0.017). Full or modified independence, i.e., FIM > 78 (Table 2), was achieved by 34 participants (58%), more frequently in the Ca than the Na group (*p* < 0.001). The median (min, max) FIM score for all 59 participants was 80 (27, 99).

### Physical activity

Physical activity was reported by 59 participants (Table 2), specifically, recreation activity by 40 (68%), exercise activity by 40 (68%), and sport activity by seven (12%) participants. More time was spent in recreation activity in the Ha group than in the Ca (*p* = 0.011) and N-a (*p* = 0.006) groups. Of those 40 who performed recreation activity, 27 also performed exercise activity (*p* = 0.944) and five also performed sport activity (*p* = 0.827). Of those who performed sport activity, all seven performed exercise activity (*p* = 0.052).

### Pain

**Fig. 5:**
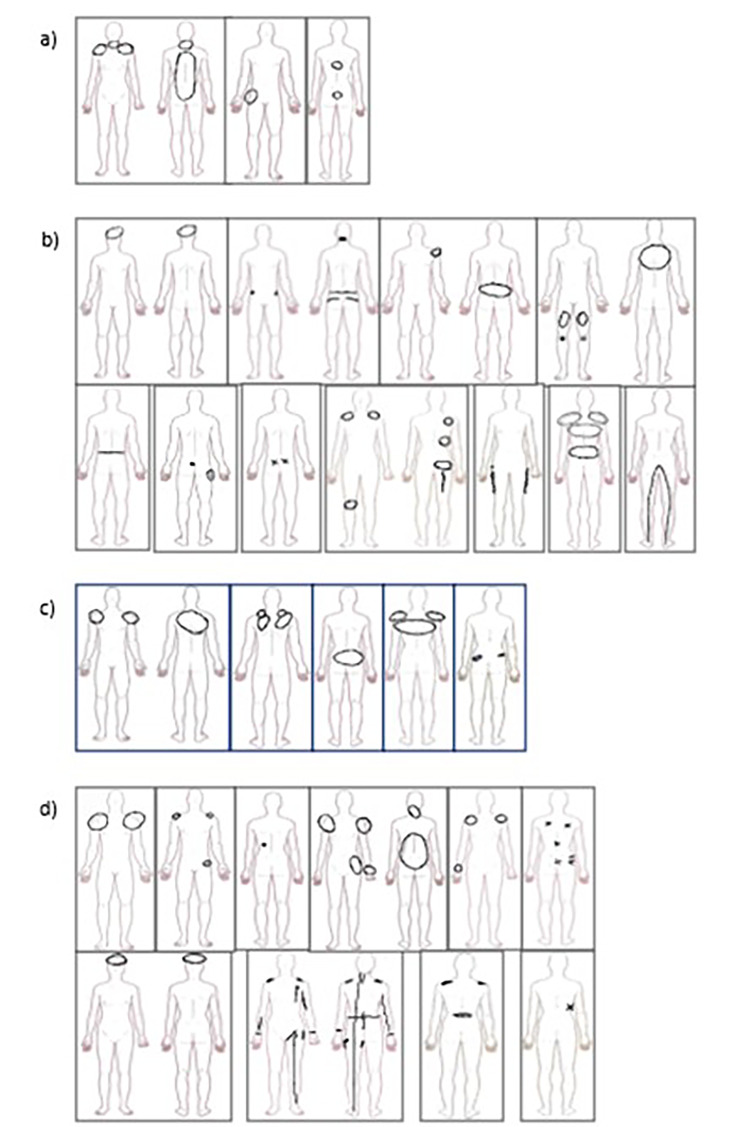
**a-d.** Pain drawings of 29 participants with respect to the ambulation groups, **(a)** Ca (community ambulation), **(b)** Ha (household ambulation), **(c)** N-f (non-functional ambulation), and **(d)** N-a (non-ambulation)

Pain is reported in Table 2. Bodily pain on the examination day and during the preceding four weeks was reported by 35/59 (57%) participants, of whom six were in the Ca, 12 in the Ha, five in the N-f, and 12 in the N-a groups. There was no difference between the ambulation groups as rated on the VAS scale. Pain was reported in the back by 20 participants (34%), in the neck/head by five (9%), in the hips by nine (15%), in the knees by three (5%), in the shoulder by 13 (22%), in the hands by two (3%), and in the arms by one participant. Pain in other locations was reported by three participants. Pain was indicated on a pre-printed drawing by 29 participants, by three in the Ca, 11 in the Ha, five in the N-f, and 10 in the N-a groups (Fig. 5a–d).

### Health status

Health status was reported by 59 participants on the dimensions of the EQ-5D-5 L (Table 4). There was no significant difference between the ambulation groups as reported on the EQ-5D-5 L VAS scale (*p* = 0.733).

**Table 4:** Percentage (number) of 59 participants by ambulation groups reporting “no” problems, “slight” problems, “moderate” problems, “severe” problems, and “unable to/extreme” problems in the EQ-5D-5 L dimensions and VAS median value

	Ambulation group			
EQ-5D-5 L dimension	Ca (*n* = 12)	Ha (*n* = 19)	N-f (*n* = 6)	Na (*n* = 22)
	% (*n*)	% (*n*)	% (*n*)	% (*n*)
**Mobility**				
no problems	25.0 (3)	26.3 (5)	0.00 (0)	0.00 (0)
slight problems	33.3 (4)	31.6 (6)	0.00 (0)	4.5 (1)
moderate problems	16.7 (2)	42.1 (8)	50.0 (3)	0.00 (0)
severe problems	0.00 (0)	0.00 (0)	0.00 (0)	0.00 (0)
unable to/extreme problems	35.0 (3)	0.00 (0)	50.0 (3)	95.5 (21)
**Self-care**				
no problems	75.0 (9)	68.4 (13)	66.7 (4)	27.3 (6)
slight problems	0.00 (0)	10.5 (2)	0.00 (0)	27.3 (6)
moderate problems	0.00 (0)	15.8 (3)	16.7 (1)	31.8 (7)
severe problems	25.0 (3)	5.3 (1)	16.7 (1)	13.6 (3)
unable to/extreme problems	0.00 (0)	0.00 (0)	0.00 (0)	0.00 (0)
**Usual activities**				
no problems	41.7 (5)	42.1 (8)	66.7 (4)	36.4 (8)
slight problems	8.3 (1)	52.6 10)	16.7 (1)	45.5 (10)
moderate problems	25.0 (3)	5.3 (1)	16.7 (1)	18.2 (4)
severe problems	0.00 (0)	0.00 (0)	0.00 (0)	0.00 (0)
unable to/extreme problems	25.0 (3)	0.00 (0)	0.00 (0)	0.00 (0)
**Pain/discomfort**				
no problems	8.3 (1)	21.1 (4)	33.3 (2)	40.9 (9)
slight problems	41.7 (5)	68.4 (13)	0.00 (0)	27.3 (6)
moderate problems	25.0 (3)	10.5 (2)	50.0 (3)	22.7 (5)
severe problems	8.3 (1)	0.00 (0)	16.7 (1)	9.1 (2)
unable to/extreme problems	16.7 (2)	0.00 (0)	0.00 (0)	0.00 (0)
**Anxiety/Depression**				
no problems	16.7 (2)	68.4 (13)	66.7 (4)	54.5 (12)
slight problems	16.7 (2)	21.1 (4)	33.3 (2)	27.3 (6)
moderate problems	50.0 (6)	10.5 (2)	0.00 (0)	13.6 (3)
severe problems	8.3 (1)	0.00 (0)	0.00 (0)	4.5 (1)
unable to/extreme problems	8.3 (1)	0.00 (0)	0.00 (0)	0.00 (0)
VAS median value (min, max)	70 (33,100)	80 (35,100)	73 (50,95)	75 (40,100)

## Discussion

This study was intended to explore function from a physical perspective in adults with MMC with a particular focus on orthosis management. All participants had been followed by the same prosthetic and orthotic provider, and largely by the same CPO, from childhood to adulthood. We were interested in exploring the functional abilities of persons provided with orthotic treatment regarded as supporting individual potential motor function from an early age and during growth [4, 5]. Regarding the heterogeneity of lesion level and the influence of additional factors on motor function even at a young age [10, 11], it is a challenge to distinguish the consequences of primary medical factors from later developing ones. In this study group, this was concretized in the variety of muscle function groups, including active quadriceps strength, even in the N-f (four of six) and N-a (three of 22) groups. This may be in accordance with Davis et al. [13], who emphasized that the effects of commonly accepted clinical factors, such as motor level, on the ambulation status of persons with MMC vary with time. To evaluate the functional status of persons with MMC, division into ambulation groups is therefore considered to match clinical reality in the most relevant way Davis et al. [13] also proposed that account should be taken of the time-varying nature of voluntarily transitioning to wheelchair use in teenage years. In the present study group, approximately 75% used a manual wheelchair and almost half used various models of powered transportation, the latter being prescribed by the healthcare regions, except in the case of one participant. Driving licences were more frequently possessed by those living in single households and by those working full- and part-time than participants of the other household status and main occupation groups. Although not a significantly differing result, this may be in line with Davis et al. [13], who noted increasing physical mobility demands in teenage years that will continue in the young adult years as well.

Use of orthoses was reported by all participants, except by two in the Ca and nine in the Na groups, a frequency higher than in another Swedish adult MMC cohort, in which most persons grew up before the active orthotic programme, which started in the early 1990s for children with MMC [41]. In a recent study of a larger Swedish MMC population, 51% in the 18–30-year age group used orthoses [3], verifying that orthosis use has become more common in that age group than in older age groups. In the study of Dicianno et al. [14], 67% reported problems with orthoses, such as skin breakdown and swelling, that affected orthosis fit, whereas only three participants reported pressure sores at the time of examination in the present study. However, as the participants were additionally asked about sores in “adult age”, approximately 62% gave positive answers, around half of whom mentioned various areas of the feet and half the buttocks region; answers that are in line with Dicianno et al. [14].

Many participants of the study group used their orthoses 10 or more hours per day. Evaluating the ease or difficulty of managing everyday functions with orthoses revealed considerable functional difficulties with respect to ambulation groups, with increasing difficulties the less ambulatory function. Regarding the significantly higher OPUS-LEFS mean score in those using KAFO-Fs than those using AFOs, i.e., less difficulties with KAFO-Fs than with AFOs, it must be recalled that eight participants in the N-a group used AFOs. To our knowledge, the OPUS-LEFS score has not previously been used in persons with MMC, so the questionnaire must be further evaluated. Concerning the question as to whether the orthosis was used for activities listed in the OPUS-LEFS, the item “Put on and take off orthopaedic assistive device” was considered not applicable. While this question does not influence the OPUS-LEFS results, it may cause concern for the participant. Since it is crucial to identify areas in which orthoses are functional for a person, this item should be removed to make the questionnaire useful for orthosis users.

As a measure of functional independence, FIM indicated similar levels in all the ambulation groups, but the more distinct independence in the Ca group than the other groups indicates lesser burden of care in participants with independent ambulatory function in the society [36]. Setting the level corresponding to full or modified independence at 78 points, it was further confirmed that the Ca group is the least dependent from the motor perspective and thus requires less assistance than the other groups. In the study by Bendt et al. [3], the mean FIM value for the entire group was 73 points, which is consistent with the present findings.

Walking velocity was assessed with the 10MWT, with participants being instructed to walk at their fastest pace while still feeling safe. The participants wearing only shoes performed the 10MWT faster than did those using AFOs and KAFO-Fs, likely because reduced muscle strength in the lower limb joints in the last two groups required orthosis use relative to muscle function level. Moreover, the increasingly observed walking velocity from Ca to Ha and N-f groups mirrors the ability of functional ambulation in the society.

Similarly, like the 10MWT, the 5STS was not performed by the N-a group. The 5STS duration was shorter in the Ca than in the Ha and N-f groups, although not significantly so. However, this finding seems relevant since the functional performance of transferring when rising from a bench without armrests requires more active hip extension strength than does walking, in which extended trunk position compensates for hip extension muscle weakness [26]. Even though most KAFO-F users were in the Ha group, the KAFOs used were free-articulated in the knee in the sagittal plane, which would not have negatively influenced the rising movement. In participants who supported themselves on the frame placed in front of them during rising, it is unclear to what extent this may have influenced the time to perform the 5STS test. The NO group had a mean time of 7.7 s compared with a mean of 9.9 s in a control group tested in the same laboratory [42]. The control persons, however, were approximately 65 years old, possibly indicating the influence of age on functional transfer performance. Further analyses will explore the kinematic aspects of rising from sitting when using orthoses. When comparing the time to perform the 10MWT versus the 5STS test, the moderate positive correlation found confirms the association between the demanding motor functions of rising to standing and fast walking in persons with MMC.

Unlike during short-distance walking, functional capacity as assessed with the self-paced 6MWT revealed that the Ca group walked farther than the Ha group. It has been reported that the heart rate continues to climb during exercise in children with disability [43], and that walking speed slows in children with MMC to maintain the heart rate at an acceptable level [11]. Compared with a control group of 25 typically developing children tested in the same laboratory [44], the mean distance on the self-paced 6MWT was 565 m, indicating similar function to that of some participants in the Ca group. Performing the 6MPT with a manual wheelchair on an oval track has shown excellent reliability in youth with SB who use wheelchairs [32]. The 6MPT was performed by 26 participants, with no significant difference between the ambulation groups, indicating that level of leg paresis plays a determinant role in variation in functional walking capacity. This may be strengthened by the finding that changes in the muscle strength of the iliopsoas and quadriceps were found to be directly related to a corresponding change in motor level [12]. Increase in physical exertion before and after the 6MWT and 6MPT as rated by the Borg scale [33], however, did not differ. This was likely influenced by adjusted velocities when both walking and wheeling. The effect on energy expenditure [6] of the energy-storing carbon-fibre spring orthoses used by 13 participants in this study remains to be investigated.

In this study, participants spent a wide range of time per week in physical activities such as recreation activities and planned exercise activities to maintain or improve physical fitness. The most frequent forms of recreation were wheelchair trips and taking walks. Exercise was reported in the form of gym workouts, horseback riding, and wheelchair activities such as land bandy, basketball, tennis, table tennis, boxing, and archery. Examples of sport activities were wheelchair land bandy, wheelchair basketball, and para ice hockey. Although no differences were found between the ambulation groups in exercise and sport participation, the Ha group was more active in recreation activities than both the Ca and N-a groups. Non-ambulatory adolescents and young adults with MMC are reportedly physically inactive, with some relationship between peak oxygen uptake and muscle strength [21, 22]. The finding that physical activity was negatively influenced by inability to walk in children with SB who were wheelchair users, particularly in older age [20], contrasted with the present finding that the N-a group reported more exercise time than did the Ca group, although less sport participation was seen to a similar degree in all groups. Sport participation, however, is suggested to be due to personal preferences rather than physical ability and could benefit from improving social support and perceived competence [23]. Since cardiovascular disease risk factors have been identified in a large proportion of adolescents and young adults with MMC [24], physical activity should be encouraged. For youth with SB who use a wheelchair for mobility or sports participation, the 6MPT has been recommended as a reliable, functional performance test of a vigorous level of exercise [32].

In accordance with Alriksson-Schmidt et al. [18] and with reports from the Swedish national MMC register, pain sites differed widely, although somewhat fewer participants reported pain in this study. By enabling the participants to express themselves subjectively by drawing the pain location, it became obvious that several body regions were involved in most participants’ pain, and in all ambulation groups, by most participants experienced back, shoulder, and neck pain. Pain should thus be actively screened for, and when present, a pain management plan for the individual be developed [18].

As could be expected, when relating the reported health status to population reference data for EQ-5D-5 L in Sweden [45] for the 30–34-year age group, apparent problems were reported in the mobility domain by the participants with MMC. Also, in the domains of self-care, usual activities, pain/discomfort, and anxiety/depression there were dissimilarities from the reference data, although somewhat fewer problems were reported in the Ca group than in the other ambulation groups. Nonetheless, the similar median values for reported current general health as rated on the EQ-5D-5 L VAS indicate similarities between the ambulation groups, as do the minimum values observed among the groups. Generally, however, there were considerable discrepancies in reported health status among the ambulation groups, which contained only small numbers of participants that may be seen as a limitation of the study. Accordingly, studies of health status in larger groups of individuals with MMC are warranted to strengthen the present findings. In the sample of Alriksson-Smith et al. [18], the mean value for the entire group was similar to that in the present study, although the major predictors of perceived health remain to be identified in the case of pain assessment [18]. The presence of intellectual disability in persons with MMC may call into question the results of self-reported outcomes, so the use of proxy answers may be necessary [18]. In this study, the cognitive level of all participants was considered sufficient to report pain and health status independently.

## Conclusion

The higher proportion of orthosis use than in other adult Swedish MMC cohorts may be explained by the intensive orthotic care provided since childhood in the present population. The results of physical function assessments in persons with MMC improve our understanding of the variation and heterogeneity in this population and shed light on the importance of individualized orthotic management. The similarities between the various ambulatory levels in terms of physical activity, pain, and health status may well mirror opportunities to achieve equal results regardless of degree of disability. Since one third of the contacted persons were unavailable for participation, the results may not be representative for the entire population receiving orthotic management by the same orthotic provider in this study. Nevertheless, a clinical implication of the study is that orthotic management is likely to be beneficial for the patient with MMC, of which the majority used their orthoses for most time of the day. Orthoses can therefore be recommended for the population of MMC, if possible, initiated in childhood. However, when assessing each individual’s functional ability, the multifactorial aspects involved in the disability of MMC should be considered.

## Data Availability

The datasets generated and analysed during the current study are not publicly available due to the large amount of motion capture data with associated Excel files; however, they are available from the corresponding author on reasonable request.

## Abbreviations

MFC: muscle function class
Ca: community ambulation
Ha: household ambulation
N-f: non-functional ambulation
N-a: non ambulation
NO: non-orthosis
FO: foot orthosis
AFO: ankle-foot orthosis
KAFO-F: knee-ankle-foot orthosis, free articulated
KAFO-L: knee-ankle-foot orthosis, locked
THKAFO: trunk-hip-knee-ankle-foot orthosis
10MWT: ten-metre walking test
6 MWT: six-minute walking test
5STS: five times sit-to-stand test
OPUS: Orthotics and Prosthetics Users’ Survey
LEFS: Lower Extremity Functional Status
